# Role of MRSI Major Metabolite Ratios in Differentiating Between Intracerebral Ring-Enhancing Neoplastic and Non-Neoplastic Lesions, High-Grade Gliomas and Metastases, and High-Grade and Low-Grade Gliomas

**DOI:** 10.7759/cureus.31841

**Published:** 2022-11-23

**Authors:** Ankush Ankush, Sanjay Sardessai

**Affiliations:** 1 Radiodiagnosis, All India Institute of Medical Sciences, Bhopal, Bhopal, IND; 2 Radiodiagnosis, Goa Medical College, Bambolim, IND

**Keywords:** brain lesions, magnetic resonance spectroscopy, non-neoplastic mass, neoplastic lesion, ring-enhancing lesions, mri spectroscopy, metabolites, mrsi

## Abstract

Introduction

The purpose of this study was to determine whether multi-voxel magnetic resonance spectroscopic imaging (MRSI) can differentiate between intracranial neoplastic and non-neoplastic and between neoplastic ring-enhancing lesions (RELs) based on differences in major metabolite ratios in their enhancing and peri-enhancing regions.

Methods

In a prospective observational study involving patients with an intracerebral RELs, MRSI using the two-dimensional multi-voxel point-resolved spectroscopy (PRESS) chemical-shift imaging (CSI) sequence at an echo time (TE) of 135 milliseconds (ms) was performed on a total of 38 patients. Of 38 lesions, 23 (60.5%) were neoplastic and 15 (39.5%) were non-neoplastic. Of the 23 neoplastic lesions, 12 were high-grade gliomas (HGGs), seven were metastases, and four were low-grade gliomas (LGGs). Major metabolite ratios, i.e., choline-to-N-acetylaspartate (Cho/NAA), choline-to-creatine (Cho/Cr), and N-acetylaspartate-to-creatine (NAA/Cr), were calculated in the enhancing and peri-enhancing regions of the RELs. A Mann-Whitney U test was run to determine differences in metabolite ratios at different voxel locations between neoplastic versus non-neoplastic lesions, HGGs versus metastatic lesions, and HGGs versus LGGs. A receiver operating characteristic (ROC) curve analysis was performed to derive cut-off values for Cho/NAA and NAA/Cr ratios in the enhancing and peri-enhancing portions of the lesions.

Results

The sensitivity, specificity, positive predictive value, and negative predictive value for categorizing an REL in either neoplastic or non-neoplastic lesions using MRSI with magnetic resonance imaging (MRI) were 91.3%, 73.3%, 84%, and 84.6%, respectively. There was a statistically significant difference between Cho/NAA (p = 0.006) and NAA/Cr (p = 0.021) ratios in the enhancing region of 23 neoplastic and 15 non-neoplastic lesions. In the voxel placed in the peri-enhancing portions, the differences between Cho/Cr ratios were just significant (p = 0.047). A cut-off score of Cho/NAA >1.67 in the enhancing regions gave a sensitivity of 82.6% and specificity of 60%. The cut-off score for NAA/Cr of <0.80 in the enhancing regions showed a sensitivity and specificity of 60.9% and 86.7%, respectively. Of the 23 neoplastic lesions, 12 HGGs and seven metastases were differentiated using the Cho/NAA ratio in the peri-enhancing region with a cut-off value of 1.21, sensitivity of 100%, and specificity of 85%. A cut-off value of Cho/Cr ≥1.45 in the peri-enhancing regions showed a sensitivity of 83% and a specificity of 71.4%. For discriminating between 12 HGGs and four LGGs both from the 23 neoplastic REL group, using the cut-off score for Cho/NAA in the enhancing portions ≥4.16 showed a sensitivity of 0.75 and specificity of 100%. In the peri-enhancing regions, a cut-off score of ≥2.07 provided a sensitivity and specificity of 83% and 100%, respectively.

Conclusion

Conventional MRI sometimes poses a diagnostic challenge in distinguishing between neoplastic and non-neoplastic lesions and other neoplastic RELs. Interpreting MRSI findings by comparing the major metabolite ratios in the enhancing and peri-enhancing regions of these lesions may enable distinction between the two.

## Introduction

The multiplanar and multiparametric acquisition capabilities make MRI the preferred modality to study intracranial space-occupying lesions (ICSOLs). Many of these ICSOLs are ring-enhancing lesions (RELs) and show ring enhancement of varying types, namely, regular or irregular, complete or incomplete, and/or thin or thick, creating a specific list of differentials to be considered given the history provided. However, in many circumstances, standard imaging is insufficient in providing enough information about the lesion to make the precise diagnosis. Magnetic resonance spectroscopy (MRS) has become today an integral part of brain MRI protocol. Proton magnetic resonance spectroscopy (1H-MRS) is a non-invasive modality that provides us with an insight into the underlying metabolic profile of these lesions.

The principal metabolites are the peaks that are consistently seen at all echo time (TE) levels, and this includes N-acetylaspartate (NAA), creatine (Cr), and choline (Cho). The NAA resonance occurs at 2.01 parts per million (ppm) and involves contributions from other N-acetyl groups such as N-acetylasparlyl glutamate (NAAG) and N-acetyl glycoproteins (NAGs). NAA is considered a marker for neuronal viability and density and is reduced in pathological conditions resulting in neuronal injury or death such as in brain tumors, strokes, neurodegenerative conditions, and multiple sclerosis [1]. A composite peak of Cho and other Cho-containing compounds (phosphocholine, phosphatidylcholine, and glycerophosphocholine) is noted at 3.2 ppm. Because phospholipids are essential precursors of myelin, Cho acts as a marker for membrane synthesis and breakdown. Thus, Cho peaks are observed in conditions of increased cellular proliferation, such as primary brain neoplasms, as well as in demyelinating diseases and infarctions because of the myelin breakdown [2]. The major Cr and phosphocreatine peak is noted at 3.03 ppm. An additional peak may be seen at 3.94 ppm. Cr is considered an indirect indicator of intracellular energy stores of the brain as it serves as a reserve for high-energy phosphates in neurons and buffers cellular ATP/ADP reservoirs [3]. The Cr concentration remains stable for each tissue type of brain and is useful as an internal reference standard. However, this is not always true as individual and regional variability is noted, especially in necrotic areas [4]. The Cr peak tends to be reduced in brain tumors, strokes, and head injury [2].

The interpretation of the MRS findings in the light of diagnostic challenge presented on review of conventional magnetic resonance (MR) imaging yields the best results. Although much of the radiology literature revolves around the qualitative interpretation of the MR spectra by radiologists in the form of the presence or absence of a specific peak, the latter probably relates to the intuitive experience of viewing conventional imaging [5]. This methodology is prone to subjective bias; however, differences in the spectral appearances at different TE intervals necessitate MRS to be performed at different TEs for reliable discrimination of these peaks. This luxury is not available in most resource-limited diagnostic departments.

This study aims to determine various cut-off values of major metabolite ratios for differentiating between neoplastic and non-neoplastic RELs using multi-voxel magnetic resonance spectroscopic imaging (MRSI) as an adjunctive modality to conventional MRI.

## Materials and methods

Study design

This prospective observational study included patients referred for MRIs to the Department of Radiology, Goa Medical College and Hospital, from December 1, 2016, to December 31, 2017. The Institutional Ethics Committee of Goa Medical College approved the study design and protocol (Reference Code GMCIEC/2016/14).

MRS was performed on a total of 61 patients, who fulfilled the inclusion and exclusion criteria. The data from 11 patients were discarded because of either poor spectroscopic data quality or as they were lost to follow-up. Data from 12 patients were excluded as only single-voxel spectroscopy (SVS) was performed on these patients. MRSI data from 38 patients were included in this study.

Inclusion and exclusion criteria

The study included patients with RELs detected on contrast-enhanced computed tomography (CECT) or clinically suspected and subsequently referred for MRI with MRS. Patients with pacemakers or implantable cardioverter-defibrillators, ferromagnetic prosthetic heart valves, aneurysm clips, non-MR compatible metal implants, and other ferromagnetic substances were excluded from the study. Before placing the patient within the gantry, informed consent was taken, and the patients were explained in vernacular language regarding intraprocedural immobilization.

Procedures and process

MRI and 1H-MRS were conducted on a 1.5 Tesla MRI MAGNETOM® Avanto, A Tim + Dot System (Siemens, Erlangen, Germany) using a dedicated head receiver coil and body radiofrequency (RF) transmitter coil.

Multiplanar multi-echo plain and post-contrast MRI of the brain was performed. We performed multi-voxel MRSI using the two-dimensional multi-voxel point-resolved spectroscopy (PRESS) chemical-shift imaging (CSI) sequence at a TE of 135 ms with a voxel size of 10.0 x 10.0 x 15.0 millimeter (mm). The volume of interest (VOI) was localized to include the lesion, the enhancing margin, and the peri-enhancing region. The peri-enhancing region was defined as white matter adjacent to and surrounding the enhancing ring, which appeared hyperintense on T2-weighted images but did not show enhancement on post-contrast T1-weighted images. VOI was adjusted to avoid the skull, scalp, cerebrospinal fluid (CSF), and air and major intracranial vessels. Six saturation bands were applied in three dimensions around the VOI. Water suppression was performed using CHESS (CHEmical Shift Selective saturation) pulses. Automatic and manual shimming was performed to adjust magnetic field inhomogeneity to obtain FWHM (full width at half maximum) of <15 Hertz (Hz).

The post-processing was performed using Syngo.MR software (Siemens, Erlangen, Germany). The following post-processing steps were carried out in an automated manner in sequence: water reference processing, Hanning filtering, zero-filling, Fourier transformation, frequency shift, baseline and phase corrections, and finally curve fitting. The voxel showing maximum metabolite concentration was chosen in MRSI. The integral value (representing the relative concentration) of each metabolite peak identified by curve fitting was recorded in the enhancing and peri-enhancing regions and the normal parenchyma. The major metabolites were those which are better demonstrated on long TE sequences (TE 135 ms), i.e., Cho which appears at 3.22 ppm, Cr at 3.02, and NAA at 2.01 ppm [6]. Poor quality spectra due to high noise-to-signal ratio, inadequate magnetic field homogeneity, and volume averaging were excluded from the study. Based on MRI and MRS findings, the diagnosis of neoplastic versus non-neoplastic lesions, HGGs versus metastatic lesions, and HGGs versus LGGs was made. Histopathological diagnosis was recorded wherever possible following stereotactic biopsy or excision of the mass lesion. Metastatic lesions were either confirmed with a known primary or on subsequent work-up for search of primary. For non-neoplastic lesions, that were not biopsied the diagnosis was confirmed by noting resolution on follow-up scans with medical management.

Statistical analysis

The statistical analysis was performed using IBM SPSS Statistics for Windows, Version 19.0. (IBM Corp., Armonk, New York). The major metabolite ratios - Cho/NAA, Cho/Cr, and NAA/Cr - were calculated. Sensitivity, specificity, positive predictive value (PPV), negative predictive value (NPV), of MRI with MRSI were calculated for differentiation of RELs into neoplastic and non-neoplastic lesions.

The median values of major metabolite ratios of neoplastic and non-neoplastic RELs were calculated and compared using the Mann-Whitney U test to test for significance at p < 0.05. The sensitivity and specificity of MRSI in the distinction between neoplastic and non-neoplastic RELs, between HGGs and metastases, and between HGGs and LGGs using the Cho/NAA, Cho/Cr and NAA/Cr ratios were estimated by analyzing the receiver operating characteristic (ROC) curve. The results are presented as percentages with 95% confidence intervals with an alpha error of 5% considered acceptable.

## Results

An MRSI study was performed on a total of 38 patients, of whom 23 (60.5%) were males and 15 (39.5%) were females. The patient's average age was 45.1 ± 17.6 years, with a range of 9 to 82 years. The mean age of patients with neoplastic lesions was 48.6 (±15.8) and that of the non-neoplastic lesions was 38.4 (±19.5). The average volume of the lesion studied was 35.8 (±35.6) cubic centimeters (cc), and the smallest lesion was 0.34 cc while the largest was 139 cc. The mean size of a neoplastic lesion was larger than that of a non-neoplastic lesion (49.1 cc versus 10.3 cc). The left parietal region (9; 23.7%) was the most common location for RELs noted in this study.

The majority of the neoplastic lesions showed a thin, irregular enhancing ring (10; 43.8%) followed by a thick, irregular enhancing rim (9; 39%). The most common enhancement pattern shown by non-neoplastic lesions was thin and regular (6; 40%), and two non-neoplastic lesions with incomplete rings were of demyelination. The highest number of RELs in the study were HGGs (12; 52%). Among the non-neoplastic lesions, tuberculoma was the most common lesion noted (7; 46.6%). Histopathological confirmation was available for a total of 22 patients including all gliomas (12 HGGs and four LGGs), one metastatic lesion, two pyogenic abscesses, and three tuberculomas. The HGGs included eight grade IV glioblastoma multiforme and four grade III anaplastic astrocytomas, while the LGGs included three cases of grade II diffuse astrocytoma and one of grade I pilocytic astrocytoma. The molecular grading was unfortunately not available. One lesion on histopathology was found to be metastatic poorly differentiated squamous carcinoma. Rest of the metastatic lesions did not undergo biopsy as were already/found to be having advanced primary malignancy (five patients with lung carcinoma and one with carcinoma breast). The two pyogenic abscesses and three tuberculomas that were biopsied showed anaerobic flora (*Bacteroides fragilis*) and caseating granulomas, respectively. Rest of the lesions were confirmed to be of non-neoplastic nature due to their presentation and conventional imaging features (two cases of demyelination and one of radiation necrosis) and on follow-up imaging after medical therapy (four cases of tuberculomas, and one each of neurocysticercosis (NCC), pyogenic, and tuberculous abscess).

To understand the diagnostic accuracy of the diagnosis made using MRI + MRSI as compared to the final diagnosis made (through biopsy or follow-up after medical therapy), 2 x 2 cross-tabulations were performed, as shown in Table 1. The MRI with MRS for differentiation of neoplastic and non-neoplastic lesions was found to be 91.3% sensitive and 73.3% specific. The PPV was 84%, and the NPV was 84.6%.

**Table 1:** MRI + MRSI diagnosis to final diagnosis cross-tabulation MRI, magnetic resonance imaging; MRSI, multi-voxel magnetic resonance spectroscopic imaging

		Final diagnosis		Total
		Neoplastic	Non-neoplastic	
MRI + MRSI diagnosis	Neoplastic	21	4	25
	Non-neoplastic	2	11	13
Total		23	15	38

On performing the Mann-Whitney U test, the integral values of NAA from voxel placed on an enhancing portion was found to be significantly different between neoplastic (median (mdn) = 2.63) and non-neoplastic (mdn = 4.4) lesions (U = 105.5, z = -2.001, p = 0.045). A significant difference was also found in values of Cho from voxels placed in the peri-enhancing region (U = 82, z = -2.703, p = 0.007). A computation of metabolite ratios was performed to evaluate differences between neoplastic and non-neoplastic lesions (Tables 2, 3). A Mann-Whitney U test was run to determine differences in metabolite ratios at different voxel locations between neoplastic and non-neoplastic RELs. There was a statistically significant difference in Cho/NAA (p = 0.006) and NAA/Cr (p = 0.021) ratios in enhancing regions of neoplastic and non-neoplastic lesions. In voxels placed in peripheral portions of the lesions, the difference between Cho/Cr was just significant (p= 0.047).

**Table 2:** Frequency distribution of ratios of metabolites in the enhancing portion on MRSI eCho/eNAA, the choline-to-N-acetyl aspartate ratio in the enhancing region; eCho/eCr, the choline-to-creatine ratio in the enhancing region; eNAA/eCr, the N-acetyl aspartate-to-creatine ratio in the enhancing region; STD, standard deviation

Final diagnosis		eCho/eNAA	eCho/eCr	eNAA/eCr
Neoplastic (n=23)	Mean	3.99	3.03	0.91
	STD	2.564	2.928	0.688
	Median	3.62	1.93	0.73
Non-neoplastic (n=15)	Mean	2.01	2.52	1.34
	STD	1.285	2.566	0.697
	Median	1.58	1.73	1.24
Total (n = 38)	Mean	3.21	2.83	1.08
	STD	2.344	2.766	0.713
	Median	2.30	1.87	0.90
	Mann-Whitney U	81	147	95.5
	Z-value	-2.733	-0.762	-2.3
	p-value	0.006	0.446	0.021

**Table 3:** Frequency distribution of ratios of metabolites in the peripheral portions on MRSI pCho/pNAA, the choline-to-N-acetyl aspartate ratio in the peri-enhancing region; pCho/pCr, the choline-to-creatine ratio in the peri-enhancing region; pNAA/pCr, the N-acetyl aspartate-to-creatine ratio in the peri-enhancing region; STD, standard deviation

Final diagnosis		pCho/pNAA	pCho/pCr	pNAA/pCr
Neoplastic (n = 2)	Mean	2.44	2.23	1.15
	STD	1.754	1.774	0.592
	Median	1.77	1.59	1.14
Non-neoplastic (n = 15)	Mean	1.13	1.50	1.45
	STD	0.423	0.487	0.593
	Median	1.09	1.51	1.19
Total (n = 38)	Mean	1.92	1.94	1.27
	STD	1.522	1.446	0.603
	Median	1.32	1.52	1.17
	Mann-Whitney U	138	106	132
	Z-value	-1.03	-1.986	-1.209
	p-value	0.3029	0.047	0.2265

**Figure 1:**
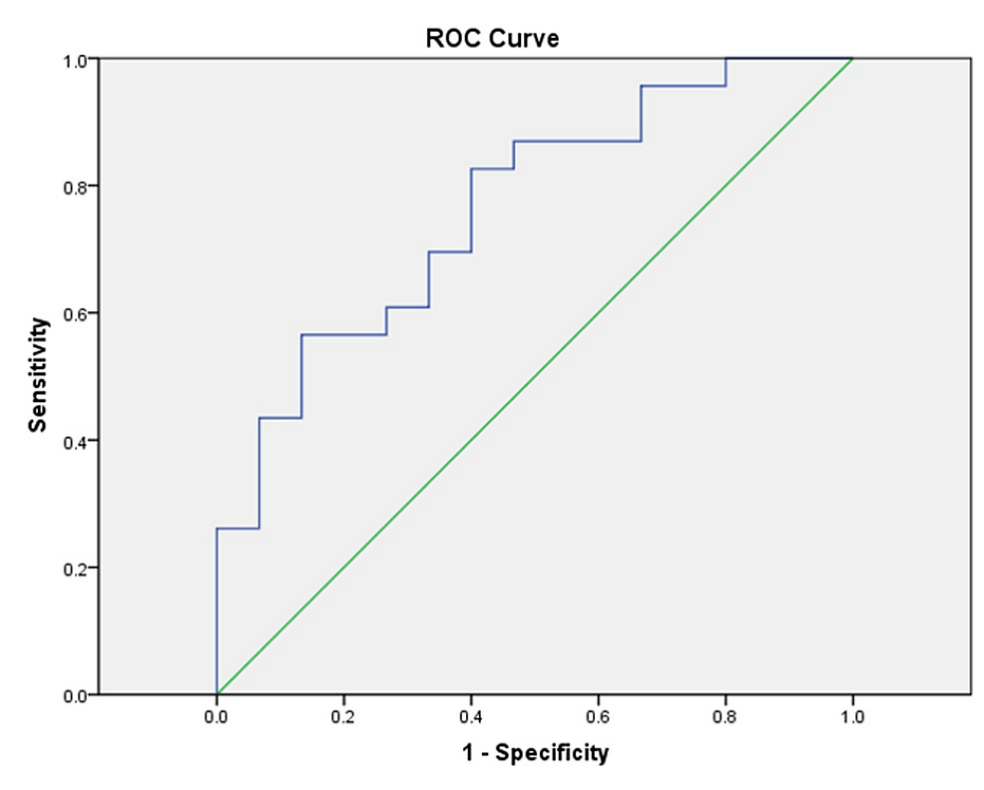
ROC curve for eCho/eNAA ROC, receiver operating characteristic; eCho/eNAA, the choline-to N-acetyl aspartate ratio in the enhancing region

ROC curve analysis was performed to derive cut-off values for eCho/eNAA ratios in the enhancing portions of the lesions (Figure 1, Table 4).

**Table 4:** The area under curve for eCho/eNAA eCho/eNAA, the choline-to-N-acetyl aspartate ratio in the enhancing region

Area	SE	p-Value	Asymptotic 95% confidence interval	
			Lower bound	Upper bound
0.765	0.078	0.006	0.613	0.917

As shown in Table 5, a cut-off score for eCho/eNAA of ≥1.67 had a sensitivity of 82.6% and specificity of 60%. A higher cut-off score, however, of 3.43 provided a greater specificity of 86.7% but a lower sensitivity of 56.5%. We performed a similar ROC curve analysis for NAA/Cr in the enhancing portions, which showed an area under the curve (AUC) of 0.723, and a cut-off value of ≤0.80 revealed a sensitivity of 60.9% and specificity of 86.7%.

**Table 5:** Coordinates of the eCho/eNAA ROC curve eCho/eNAA, the choline to N-acetyl aspartate ratio in the enhancing region

Positive if > or =	Sensitivity	1 - Specificity	Specificity	Youden index
1.629808	0.826	0.467	0.533	0.359
1.667308	0.826	0.400	0.600	0.426
1.761232	0.783	0.400	0.600	0.383
3.044192	0.565	0.200	0.800	0.365
3.425803	0.565	0.133	0.867	0.432
3.557515	0.522	0.133	0.867	0.388

In an attempt to further study differences in metabolite ratios in discriminating between different neoplastic lesions, there were statistically significant differences noted between all ratios in the peri-enhancing regions and only of Cho/NAA ratio in the enhancing region between 12 HGGs and seven metastatic lesions (Table 6). To determine cut-off values for discriminating between HGGs and metastases, we performed a ROC curve analysis and found a cut-off value of Cho/Cr ≥1.45 in the peri-enhancing regions to provide a sensitivity of 83% and specificity of 71.4%. The Cho/NAA ratio in the peri-enhancing region provided a cut-off value of 1.21; the sensitivity was 100% and the specificity was 85%. In the ROC analysis, the AUC for Cho/Cr was 0.857 and that for Cho/NAA was 0.964.

**Table 6:** Comparison of the differences in metabolite ratios between high-grade gliomas vs. metastases eCho/eNAA, the choline-to-N-acetyl aspartate ratio in the enhancing region; eCho/eCr, the choline-to-creatine ratio in the enhancing region; eNAA/eCr, the N-acetyl aspartate-to-creatine ratio in the enhancing region; pCho/pNAA, choline-to-N-acetyl aspartate ratio in the peri-enhancing region; pCho/pCr, choline-to-creatine ratio in the peri-enhancing region; pNAA/pCr: N-acetyl aspartate-to-creatine ratio in the peri-enhancing region

	eCho/eNAA	eCho/eCr	eNAA/eCr	pCho/pNAA	pCho/pCr	pNAA/pCr
Mann-Whitney U	3.000	21.000	27.000	3.000	12.000	10.000
Z-value	-3.296	-1.775	-1.268	-3.296	-2.535	-2.704
p-value	0.001	0.076	0.205	0.001	0.011	0.007

When comparing metabolite ratios between 12 HGGs and four LGGs, statistically significant differences were noted only in Cho/NAA ratio in both enhancing and peri-enhancing regions (Table 7). Furthermore, ROC curve analysis to determine cut-offs for discriminating between high-grade and low-grade gliomas revealed a ratio of ≥4.16 of Cho/NAA in the enhancing portions with a sensitivity of 0.75 and specificity of 100%, and in the peri-enhancing regions a cut-off value of ≥2.07 provided a sensitivity of 83% and specificity of 100%.

**Table 7:** Comparison of the differences in metabolite ratios between high-grade versus low-grade gliomas eCho/eNAA, choline-to-N-acetyl aspartate ratio in the enhancing region; eCho/eCr, choline-to-creatine ratio in the enhancing region; eNAA/eCr: N-acetyl aspartate-to-creatine ratio in the enhancing region, pCho/pNAA, choline-to-N-acetyl aspartate ratio in the peri-enhancing region; pCho/pCr, choline-to-creatine ratio in the peri-enhancing region; pNAA/pCr, N-acetyl aspartate-to-creatine ratio in the peri-enhancing region

	eCho/eNAA	eCho/eCr	eNAA/eCr	pCho/pNAA	pCho/pCr	pNAA/pCr
Mann-Whitney U	3.000	17.000	11.000	2.000	9.000	10.000
Z-value	-2.547	-0.849	-1.576	-2.668	-1.819	-1.698
p-value	0.011	0.396	0.115	0.008	0.069	0.090

The various metabolite ratios of non-neoplastic lesions are presented in Table 8. On comparing major metabolite ratios of these lesions, we did not observe any statistically significant differences between them.

**Table 8:** Metabolite ratios of non-neoplastic lesions eCho/eNAA, choline-to-N-acetyl aspartate ratio in the enhancing region; eCho/eCr, choline-to-creatine ratio in the enhancing region; eNAA/eCr, N-acetyl aspartate-to-creatine ratio in the enhancing region; pCho/pNAA, choline-to-N-acetyl aspartate ratio in the peri-enhancing region; pCho/pCr, choline-to-creatine ratio in the peri-enhancing region; pNAA/pCr, N-acetyl aspartate-to-creatine ratio in the peri-enhancing region; RELs, ring-enhancing lesions; STD, standard deviation

Non-neoplastic RELs		eCho/eNAA	eCho/eCr	eNAA/eCr	pCho/pNAA	pCho/pCr	pNAA/pCr
Demyelination (n=2)	Mean	1.54	1.67	1.08	1.24	2.02	1.55
	STD	0.057	0.278	0.221	0.479	1.291	0.445
	Median	1.54	1.67	1.08	1.24	2.02	1.55
Neurocysticercosis (n=1)	Mean	0.59	0.97	1.66	0.58	1.24	2.15
	STD	-	-	-	-	-	-
	Median	0.59	0.97	1.66	0.58	1.24	2.15
Pyogenic abscess (n=3)	Mean	2.06	2.66	1.35	1.05	1.48	1.69
	STD	0.905	1.414	0.474	0.599	0.402	0.986
	Median	2.43	1.96	1.57	0.92	1.51	1.15
Radiation necrosis (n=1)	Mean	0.83	1.25	1.50	0.86	1.26	1.15
	STD	-	-	-	-	-	-
	Median	0.83	1.25	1.50	0.86	1.26	1.15
Tuberculous abscess (n=1)	Mean	1.87	1.64	0.88	1.17	1.52	1.30
	STD	-	-	-	-	-	-
	Median	1.87	1.64	0.88	1.17	1.52	1.30
Tuberculoma (n=7)	Mean	2.51	3.24	1.40	1.21	1.43	1.29
	STD	1.623	3.610	0.985	0.440	0.322	0.549
	Median	1.63	2.024	1.24	1.09	1.55	1.09
Total (n=15)	Mean	2.01	2.52	1.34	1.13	1.50	1.45
	STD	1.285	2.566	0.697	0.423	0.487	0.593
	Median	1.58	1.73	1.24	1.09	1.51	1.19

## Discussion

The high sensitivity of MRS in conjunction with conventional imaging to differentiate between neoplastic and non-neoplastic lesions has been previously mentioned by other authors [7-10] and has been revalidated by the present study. Alam et al. [11], in a similar study of 78 histopathology-proven RELs, found the sensitivity, specificity, PPV, and NPV of MRS to be 90.16%, 64.70%, 90.16%, and 64.70%, respectively, when used to discriminate between neoplastic and non-neoplastic lesions. Lai et al. [12], in a study of 50 patients with intracranial cystic lesions found sensitivity, specificity, PPV, and NPV to be 95.2%, 100%, 100%, and 95.8%, respectively, when using MRS with conventional MRI and diffusion-weighted imaging (DWI).

**Figure 2:**
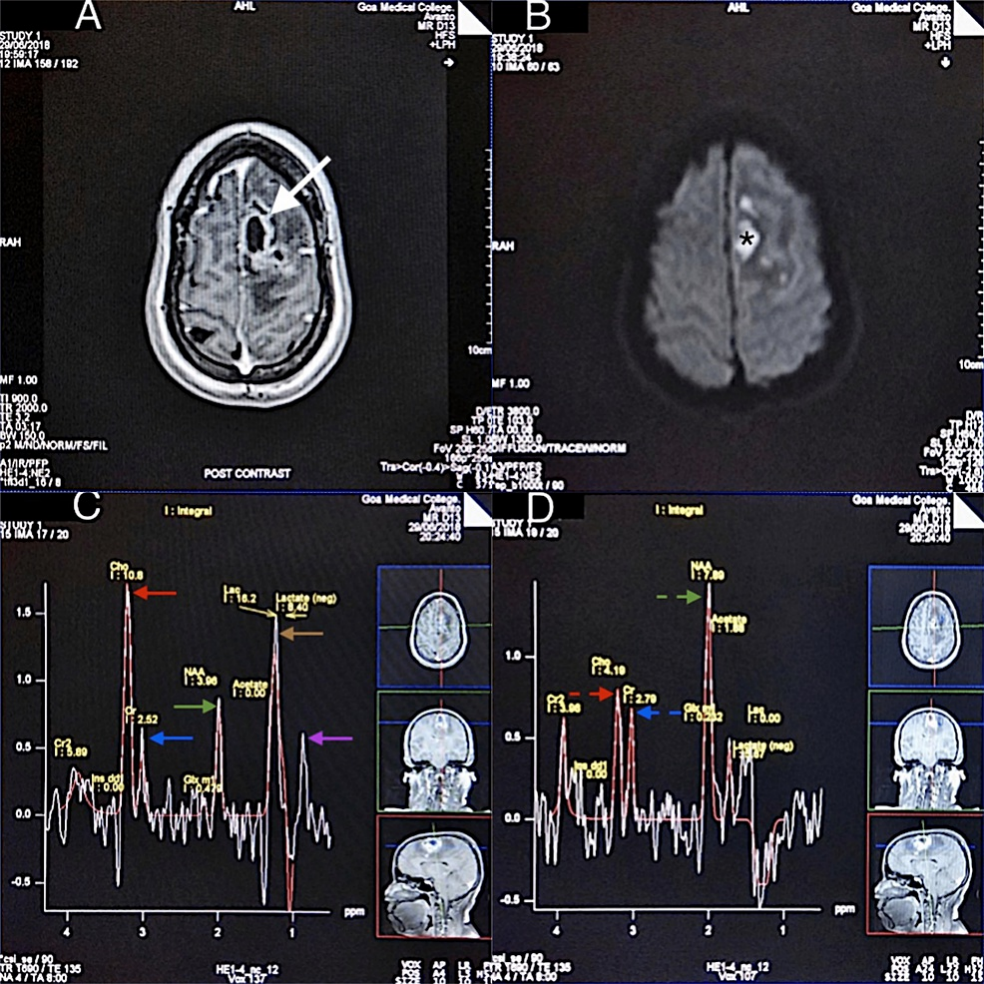
An MRI and MRSI evaluation of a pyogenic abscess case (A) An axial T1-weighted, post-contrast MRI shows a thick-walled, peripherally enhancing lesion in the left frontal lobe (white arrow). (B) The DWI shows the central portion of the lesion showing restricted diffusion (*). (C) The MRSI at TE=135 with the voxel in an enhancing region shows elevation of the lipid/lactate peak at 1.33 ppm (brown arrow) and an increased amino-acid peak (valine, leucine, and isoleucine) at 0.9 ppm (violet arrow). (D) A voxel in the peri-enhancing region shows a reduced Cho/Cr (red and blue dashed arrows) and Cho/NAA ratio (red and green dashed arrows) as compared to enhancing region (Cho, Cr, and NAA marked with red, blue, and green arrows, respectively in Figure 2C). MRI, magnetic resonance imaging; MRSI, multi-voxel magnetic resonance spectroscopic imaging; DWI, diffusion-weighted imaging; NAA, N-acetyl aspartate; Cho, choline; Cr: creatine

An increase in Cho levels indicates increased cellular membrane and myelin turnover. The median Cho levels in the enhancing portions of the lesions were higher in neoplastic lesions (mdn = 9.45) than in the non-neoplastic lesions (mdn=6.4), but the difference was only borderline significant. Significant differences were also noted in NAA values in the enhancing portions, with a lower median value noted in the neoplastic lesions (2.63 vs 4.4). This resulted in the calculated ratios of Cho/NAA and NAA/Cr in the enhancing portions being statistically different between neoplastic and non-neoplastic lesions, as noted in Table 2. This underlines the importance of calculating metabolite ratios rather than the absolute integral values of metabolites. A Cho/NAA ratio of more than one has been generally considered as indicative of a neoplastic lesion [13]. Aydin et al. [14], in a study of 33 patients, found in the tumor group similar average values of 2.42, 0.97, and 2.42 for Cho/NAA, NAA/Cr, and Cho/Cr, respectively, which were significantly different from the non-neoplastic group. Similarly, Majos et al. [15], in a retrospective analysis of 84 cases, found a Cho/NAA ratio of more than 1.9 at long TE as a classifier with a diagnostic accuracy of 79%. Ferraz-Filho et al. [16], in a study of 81 patients, provided a discriminatory boundary of Cho/Cr > 1.97 and NAA/Cr < 1.12 for differentiation between inflammatory brain lesions and high-grade neoplasms. These findings are consistent with our data; however, we did not observe any statistically significant difference between the Cho/Cr ratio in the enhancing portions. There was, though, barely a statistical difference in the peri-enhancing region (p = 0.047) (Table 3 and Figure 2). This is probably due to the peri-enhancing edematous regions in neoplastic lesions; for example, HGGs represent a combination of vasogenic edema and neoplastic cell infiltration, unlike non-neoplastic lesions.

To determine cut-off scores, an ROC curve analysis of the Cho/NAA ratio in our study showed findings similar to a study by Alam et al. mentioned earlier in the present paper, which reported a cut-off score of Cho/NAA = 2.55, which showed a sensitivity of 70% in differentiating between neoplastic and non-neoplastic lesions [11]. The value in Rand et al.’s study of 53 patients showed the area under the aggregate ROC curve in the blinded discrimination of neoplasm from non-neoplasm to be 0.89. However, this study was based on SVS [17].

**Figure 3:**
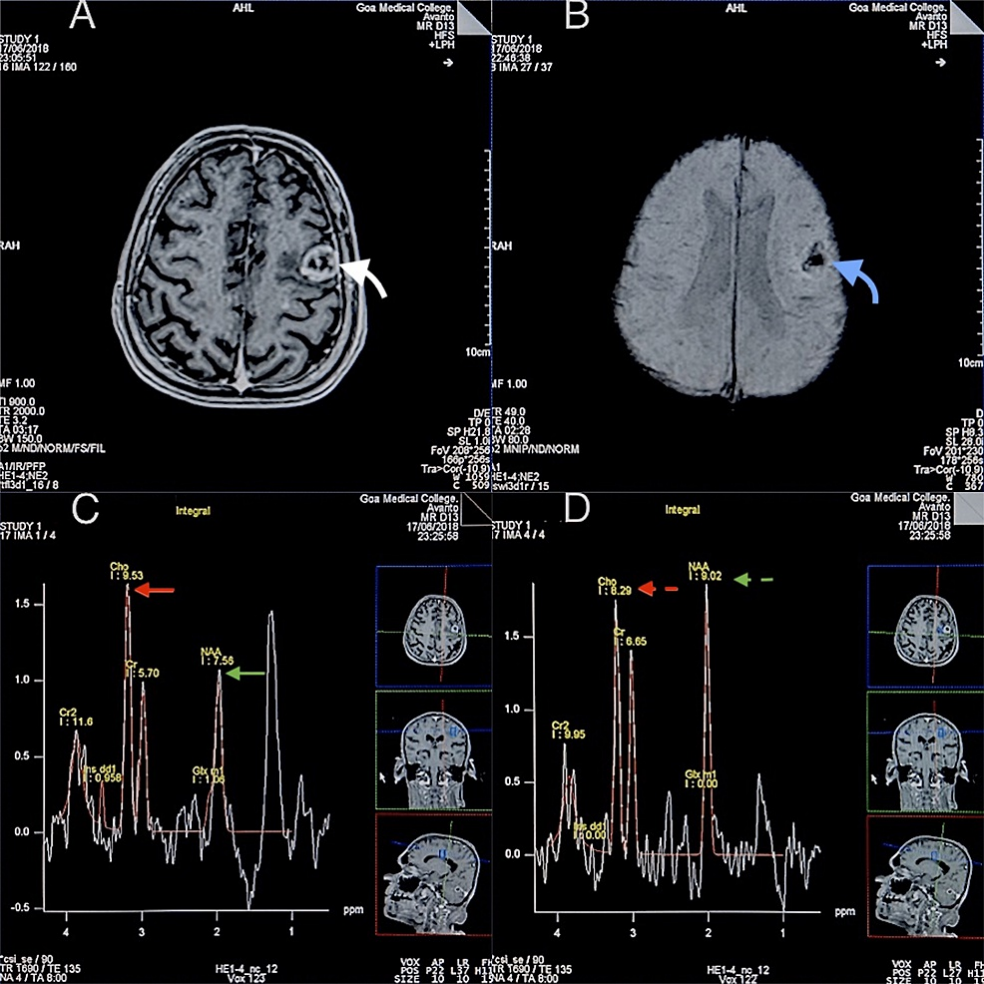
An MRI and MRSI evaluation of a case of metastases to the brain (A) An axial T1-weighted, post-contrast MRI shows a thick-walled, peripherally enhancing lesion in the left frontal lobe (curved white arrow). (B) An SWI showing a central area of "blooming" suggesting hemorrhage (curved blue arrow). (C and D) An MRSI at TE=135 with a voxel in the enhancing region (C) shows an elevated Cho/NAA ratio (red and green arrows) as compared to a voxel in the peri-enhancing region (D) (red and green dashed arrows). MRI, magnetic resonance imaging; MRSI, multi-voxel magnetic resonance spectroscopic imaging; SWI, susceptibility-weighted imaging; NAA, N-acetyl aspartate; Cho, choline

When comparing major metabolite ratios in the peri-enhancing regions of HGGs and metastases, there were significant differences in Cho/NAA, Cho/Cr, and NAA/Cr ratios noted in the peri-enhancing regions. This is likely because in the peri-enhancing regions of the metastatic lesions there are no tumor cells or vascular endothelial proliferation, rather almost purely vasogenic edema due to leakage of plasma fluid from altered tumor capillaries (Figure 3). These findings are similar to the result of Law et al., which found Cho/Cr in the peritumoral region (2.28 ± 1.24) of gliomas to be statistically different than that of metastases (0.76 ± 0.23) [18]. Our findings are also consistent with a study performed by Server et al. on 53 patients with high-grade gliomas and 20 patients with metastatic tumor in which they found significant differences in the peritumoral Cho/Cr, Cho/NAA, and NAA/ Cr ratios [19]. In the same study, they also proposed a cut-off value of 1.24 for peritumoral Cho/Cr ratio to provide sensitivity and specificity of 100% and 88.9% and a cut-off value of 1.11 for peritumoral Cho/NAA ratio; the sensitivity was 100% and the specificity was 91.1%. Wang et al. published a pooled quantitative synthesis of seven studies consisting of 261 patients to differentiate high-grade gliomas from metastasis [20]. They concluded that the Cho/NAA ratio in the peritumoral region improves the diagnostic accuracy of MRSI in differentiating high-grade glioma from metastasis as this ratio showed higher specificity and AUC than Cho/Cr. Our findings, as noted above, were consistent with this observation.

**Figure 4:**
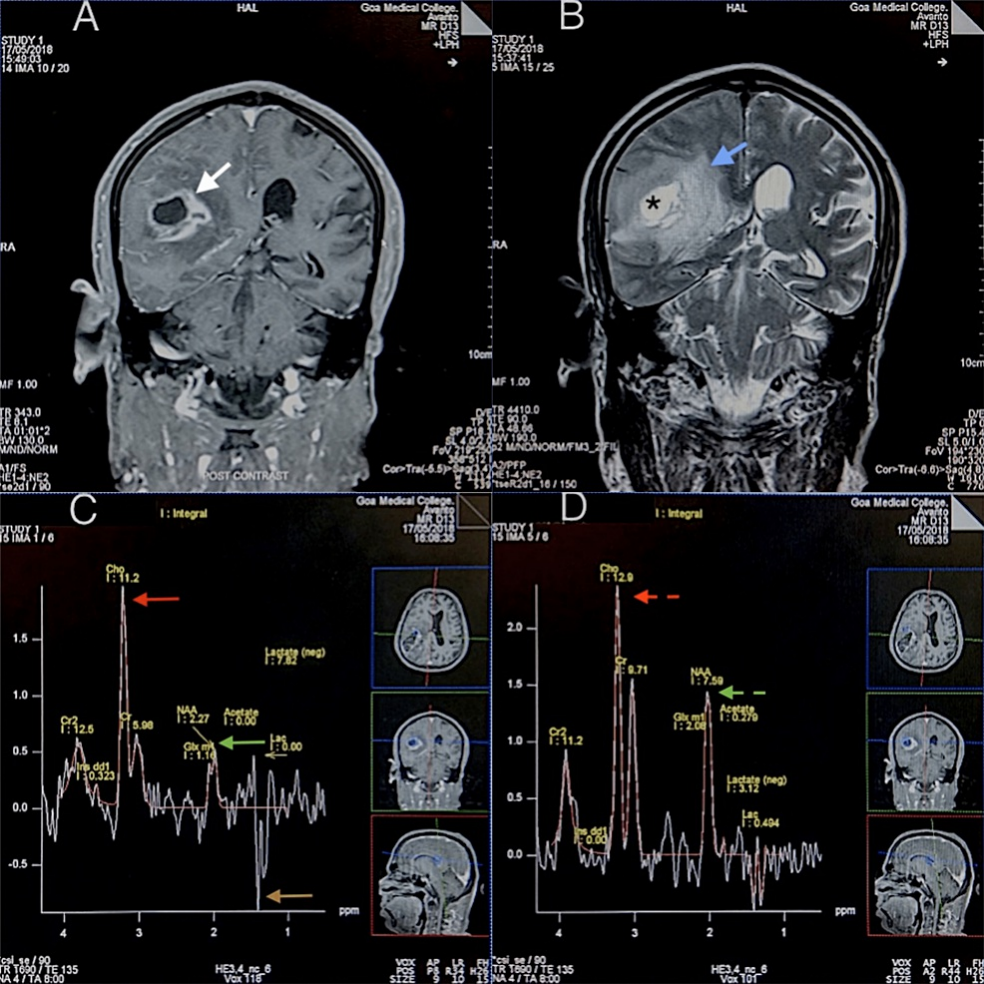
An MRI and MRSI evaluation of a high-grade glioma case (A) A coronal T1-weighted, post-contrast MRI showing an HGG with thick, irregular peripheral enhancing rim (white arrow). (B) A coronal T2-weighted MRI shows central hyperintensity suggestive of necrosis (*) with surrounding perilesional edema (blue arrow). (C) An MRSI at TE=135 with a voxel in the enhancing region showing a prominent Cho peak with a small NAA peak (red and green arrows respectively). There is an inverted lactate peak (brown arrow) as would be expected at this intermediate echo time. (D) A voxel in the peri-enhancing region shows a prominent Cho peak (red dashed arrow); however, a reduced Cho/NAA ratio (red and green dashed arrows) is seen as compared to enhancing region. MRI, magnetic resonance imaging; HGG, high-grade glioma; MRSI, multi-voxel magnetic resonance spectroscopic imaging; NAA, N-acetyl aspartate; Cho, choline

In comparison of HGGs and LGGs, a statistical difference was noted only between the Cho/NAA ratio in the enhancing and the peri-enhancing regions (Figure 4). According to a systematic review and meta-analysis published in 2016 that included 30 articles and 1,228 patients evaluating the diagnostic performance of MRS in differentiating high-grade gliomas from low-grade gliomas, the Cho/NAA ratio had greater sensitivity and specificity than the Cho/Cr and NAA/Cr ratios despite the fact that the AUC between the Cho/Cr and Cho/NAA groups was not significantly different [21]. The AUC for Cho/NAA reported in this meta-analysis was 0.87. The AUC in our study was 0.938 in the enhancing regions and 0.958 in the peri-enhancing regions, revalidating these findings.

We also compared various non-neoplastic lesions for differences in major metabolite ratios using MRSI and did not observe any significant differences. We noted that Cho/Cr ratio in the enhancing region of a case of NCC was 0.97, and the mean value of Cho/Cr ratio in the enhancing region of tuberculomas was 3.24. This finding is consistent with the study of Pretell et al., which found that the Cho/Cr ratio was greater than 1 in all tuberculomas but in none of the cysticerci [22]. They also found tuberculomas to have lower NAA values, which was also found in our study (NAA/Cr = 1.66 in NCC vs. 0.88 in tuberculomas). However, their research used SVS, which may prove to be a better method for studying tiny lesions such as NCC. Lastly, these findings should be seen in light of the fact that our study had a sample size constraint of the non-neoplastic lesions.

MRSI offers distinct advantages in studying RELs compared to a single-voxel study. As multiple voxels are studied simultaneously, it allows the examination of the metabolic profiles of the lesion core, enhancing rim, peri-enhancing region, and surrounding normal brain parenchyma (including the contralateral hemisphere). As most neoplastic lesions have a heterogeneous metabolic profile, the high spatial resolution offered by MRSI is advantageous as it allows those regions with the most marked metabolic abnormalities to be chosen for analysis. Unlike SVS, MRSI is also less susceptible to partial volume effects from the surrounding normal brain parenchyma, CSF, and necrotic regions of RELs. All RELs are not amenable for MRSI study; these include RELs located in the posterior fossa and at cortical locations, which present with variable field homogeneity due to magnetic susceptibility effects. The RELs that are predominantly hemorrhagic (such as metastasis) and RELs that are significantly smaller than the MRSI voxel size may not offer a characteristic spectrum for analysis. An MRSI study itself takes 15-20 minutes, and despite using a reduced field of view to obtain a good-quality spectrum, many patients may not cooperate for the total duration of the study (conventional MRI and MRSI) and may require sedation.

## Conclusions

The study suggests that the major metabolite ratios Cho-to-NAA, Cho-to-Cr, and NAA-to-Cr calculated in the enhancing and peri-enhancing regions of the ring-enhancing lesions are useful as an adjunctive technique to improve diagnostic confidence in cases with equivocal conventional MRI findings. This may obviate the need for a brain biopsy and is especially useful in biopsy inaccessible regions such as the brainstem and eloquent cortex. However, if a brain biopsy is required, MRS also aids to define the lesion margins, especially in cases of neoplastic lesions based on elevated levels of the Cho-to-NAA ratio. Nonetheless, it should be understood that not all ring-enhancing lesions may be amenable to a multi-voxel spectroscopy study due to technical issues such as lesion location and patient cooperation. Further studies with higher sample sizes or meta-analyses are suggested to better understand the differences between metabolite ratios of non-neoplastic lesions.
